# Anatomical Study of the Scleral Ring and Scleral Ossicles of the Ostrich (*Struthio camelus*) With Gross Anatomical Methods and Diagnostic Imaging Techniques

**DOI:** 10.1155/vmi/4993179

**Published:** 2025-07-01

**Authors:** Majid Masoudifard, Omid Zehtabvar, Arman Shahbazi, Soroush Bakhtiari Raad, Seyyed Hossein Modarres Tonekabony

**Affiliations:** ^1^Department of Surgery and Radiology, Faculty of Veterinary Medicine, University of Tehran, Tehran, Iran; ^2^Anatomy Sector, Department of Basic Sciences, Faculty of Veterinary Medicine, University of Tehran, Tehran, Iran; ^3^Faculty of Veterinary Medicine, University of Tehran, Tehran, Iran; ^4^Department of Basic Sciences, Faculty of Veterinary Medicine, Islamic Azad University, Science and Research Branch, Tehran, Iran

**Keywords:** anatomy, avian, micro-CT scan, ossicle, ostrich, scleral ring

## Abstract

The ostrich is a type of flightless bird native to Africa and a scavenger, belonging to the Struthionidae family. The eyeball in birds has more unique features. One of these features is the presence of a scleral ring. The intrascleral bones that surround the eye are in the form of a group of bones with different shapes, which form the scleral ring. It helps the eyeball against trauma. The head of 5 adult male ostriches was examined in this study. According to the results, the scleral ring in *Struthio camelus* has anterior and posterior parts and the lens is in the immediate vicinity of the anterior part. The right and left scleral rings and eyeballs are bilaterally symmetrical in terms of the shape and size but the number of ossicles can be different. This structure consisted of 15–17 ossicles and all the rings are Type A. The shape of the scleral ring and its constituent parts is one of the characteristics used for the classification of animals.

## 1. Introduction

The ostrich is native to Africa [1]. This bird is a member of the Struthionidae family, the genus *Struthio* and the species *Struthio camelus*[2]. In certain nations, beginning in 1980, an ostrich industry was established, focusing on the production of meat, leather, and feathers [3].

The eye structure of avians is very similar to mammals. Notably, the proportion of the eyeball in relation to the size of the head exceeds that of mammals [4]. Additionally, the globe, which occupies a significant portion of the orbital cavity, exhibits less mobility compared to mammalian equivalent [5]. It is worth noting that in the majority of avian species, the weight of the ocular organs surpasses that of the brain [4].

Among vertebrates, the ostrich possesses the largest eye with a diameter of 50 mm, which is twice the size of the human eye, and in some birds such as owls, the eyeball is bigger than humans [4, 6]. The avian eye, although varying in size, also displays substantial variation in shape. However, a common feature is that the posterior region of the globe, which is nearly hemispheric, is disproportionately larger than the anterior segment [7]. This intermediate region, connecting the anterior and posterior segments, exhibits a variable shape based on the scleral ossicles [8].

In birds, the posterior segment of the fibrous tunic of the eye is known as the sclera, which contains hyaline cartilage and bone. This cartilage extends to the point where the scleral ossicles are located. Scleral ossicles are dermal bones that exist in the eyes of various vertebrates [9]. Arranged in the form of plates or ossicles, the bones within the sclera form the sclerotic ring. These ossicles are placed together by cartilaginous joints and are not connected to the rest of the skull. The number of scleral ossicles can vary from 10 to 18, but most avian species possess around 14 to 15 [4, 10].

The encircling of the eye by a ring is induced by a series of conjunctival papillae that appear a few days earlier. These papillae, known as scleral papillae [11, 12], are transient structures that develop above the presumptive ossicles and stimulate ossicles in the ectomesenchyme below. The scleral cartilage, conversely, is stimulated by the retinal pigmented epithelium [13–15].

While the individual role of each ossicle is still debated, two collective functions are well established. The first is to protect and support the eyeball during flying or diving, and the second is to assist the function of the ciliary muscles, particularly in the anterior part of the cornea, suggesting a role in visual accommodation [4, 16–18].

The development of ossicles in a remarkably synchronized manner corresponds precisely with the progression of conjunctival (scleral) papillae development. Coulombre et al. discovered a direct correlation between scleral ossicles and conjunctival papillae, with each papilla developing directly above a single ossicle and subsequently disappearing after induction. The prevention of ossicle development occurs when one papilla is removed [19].

Hall and Miyake elucidate that scleral papillae ossify through intramembranous ossification in reptiles and birds, while fish undergo endochondral ossification. Experimental investigations on *Gallus gallus* embryos have demonstrated that the scleral bone structure arises from the condensations of mesenchymal cells originating from the neural crest [20–23].

Certain vertebrates, such as fish, reptiles, and birds, possess both bone and cartilage elements, while others, like crocodilians and ophidians, solely possess scleral cartilage [23, 24]. The occurrence of scleral ring is observed in numerous reptiles, excluding crocodiles and snakes, as well as dinosaurs, and the number of ossicles in reptiles surpasses that of birds [25, 26].

The morphology, quantity, and distribution of the scleral ossicles exhibit significant variation across distinct animal groups [27]. Among most avian species, these ossicles possess a rectangular configuration, while in some cases, they demonstrate a slightly elongated and concave shape [21, 28].

Ossicles that undergo alterations in their coverage pattern are referred to as excellent ossicles, which can be classified into two types: plus and minus. If both sides of an ossicle are covered by their preceding and subsequent ossicles, it is categorized as plus. Conversely, if both sides of an ossicle are covered by the preossicles and postossicles, it is referred to as minus. An ossicle is deemed interlocked or imbricated if one side overlaps with the preceding ossicle and the other side overlaps with the subsequent ossicle. In avian species, the scleral ring is divided into two types (A and B) based on the number of excellent ossicles present. Type A consists of four excellent ossicles, while Type B possesses two excellent ossicles. Type B is commonly observed in owls and birds of prey. It should be emphasized that these characteristics may vary across different species. Furthermore, it is important to note that the number of ossicles in the right and left rings of a bird may differ from each other [29].

We adhered to the conventions established by Lemmrich when numbering the ossicles and describing the patterns of ossicle overlap. Lemmrich emphasized that in Type A rings, the most dorsal ossicle always overlaps with both of its neighboring ossicles, which he referred to as “+” elements. Opposite this dorsal “+” element, there is another “+” element designated as ossicle number 1. The counting of ossicles around the ring is done in a clockwise direction on the right eye scleral ring and counterclockwise on the left scleral ring, starting from this ventral “+” ossicle. In Lemmrich's Type B pattern, there is only a single ventral plus element and a single dorsal minus element present [30].

Diagnostic imaging techniques, such as radiography, computed tomography (CT) scan, and micro-CT Scan, are highly valuable not only in veterinary clinics for making diagnoses but also for anatomical studies. These techniques are frequently employed by anatomists to investigate anatomical structures in live animals.

Micro-CT systems, also known as high-resolution X-ray CT systems, have been developed and utilized successfully in small animal studies over the past two decades. These systems, which are designed specifically for high-resolution imaging, are based on the same physical principle as clinical CT scanners. By capturing hundreds of two-dimensional (2D) projections from various angles around the animal, a micro-CT scanner generates three-dimensional (3D) tomographic data at microscopic resolution (with a voxel size of ≤ 100 μm^3^) [31]. The initial applications of micro-CT in small animal imaging focused on bone imaging, as demonstrated by studies conducted by Feldkamp et al. [32] and Kinney et al. [33]. This technique is particularly suitable for bone imaging due to the inherent contrast between bone and soft tissues, which arises from the greater effective atomic weight of bone. Consequently, micro-CT enables noninvasive and high-resolution bone imaging without the need for an exogenous contrast agent. Moreover, it allows for accurate quantification of various bone parameters, such as cross-sectional area, cortical thickness, bone mineral density, bone volume, bone surface ratio, and trabecular thickness [34].

So far, not many studies have been done on the structure of the eyeball and scleral ring in ostriches. This study was carried out with the aim of examining the scleral ring in ostrich. In addition, the pattern, shape, size, and number of ossicles in *Struthio camelus* have been studied.

## 2. Materials and Methods

Five heads of adult male ostrich were included in this study, and the average weight of these ostriches was 111 ± 3 kg. The ostriches belonged to Tehran Province in Iran.

### 2.1. Radiographic Studies

Radiographic images were done with using a digital radiography system (Kodak Directview CR 850) after positioning the heads in a dorsoventral manner, with technical parameters set at 30 mAs and 130 Kvp to ensure optimal imaging quality.

### 2.2. CT Scanning

**Figure 1:**
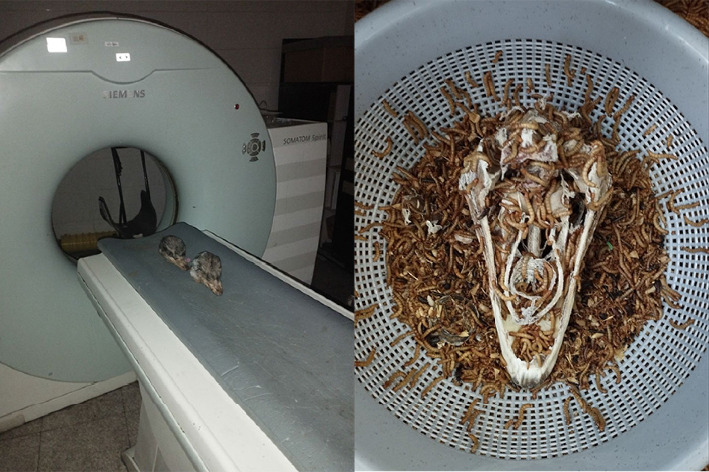
Images of the steps of performing CT scan imaging and bone separation.

The heads of all ostriches were transferred to the radiology department of the Small Animal Hospital in the Faculty of Veterinary Medicine at the University of Tehran in Iran. Subsequently, a CT scan was conducted for all subjects. The ostrich heads were positioned in ventral recumbency, and all scans were obtained using a two-detector scanner (Siemens Somatom Spirit/Germany) aligned vertically with the longitudinal axes of the animal. The imaging protocol for this procedure included the following technical parameters: rotation time of 1 s, slice thickness of 1 mm, reconstruction interval of 0.5–1 mm, pitch of 1, X-ray tube potential of 130 kV, and X-ray tube current of 144 mAs. For each segment, the appropriate window width (WW) and window level (WL) were chosen for the section, as indicated in each of the CT scan images. The bone window was utilized for image examination. Additionally, 3D images were reconstructed using the Osseous-Shaded-vp pattern. The Syngo MMWPVE40A software was employed for the 3D reconstruction of digital images (Figure 1).

### 2.3. Micro-CT Scan Examination

The eye ball was separated from the skull and prepared for micro-CT scan examination. In this study, we used an in vivo X-ray micro-CT scanner (LOTUS inVivo, Behin Negareh Co., Tehran, Iran) at the Preclinical Core Facility (TPCF) based at Tehran University of Medical Sciences. LOTUS-inVivo has a cone beam micro-focus X-ray source and a flat panel detector. In order to obtain best possible image quality, the X-ray tube voltage and its current were set to 70 kV and 70 μA, respectively, and frame exposure time was set to 1 s by 3 magnification. Total scan duration was 30 min. Slice thicknesses of reconstructed images were set to 25 μm. The entirety of the protocol configuration procedures was managed by the LOTUS-invivo software. The obtained 3D data were reconstructed utilizing LOTUS inVivo-REC through the application of a standardized Feldkamp, Davis, and Kress (FDK) algorithm.

### 2.4. Gross Anatomical Studies

An anatomic analysis was conducted based on the information obtained in the preceding stage and information accessible on other avian species. Initially, the skin and unnecessary tissues were eliminated, followed by the maceration of all osseous structures using the mealworm technique (*Tenebrio molitor*) for a duration of 4 weeks. This process was carried out under specific conditions, maintaining a temperature of 21°C and a relative humidity of 70%, in order to create an optimal environment for insects. Following the completion of ossicle cleaning, the specimens were transferred to an Olympus SZX12 stereo microscope, which was equipped with the ASP-Cell Pad E digital camera. This facilitated further investigation and acquisition of data (Figure 1).

### 2.5. Morphometric Studies

Morphometric measurement using digital CT images was conducted using the Syngo MMWPVE40A software. Volumetric measurements were performed using a selected Hounsfield unit range of −120 to +1900, which represents soft tissue and bone. The comparison of parameters between the right and left eyeballs was carried out by running a paired sample *t*-test in SPSS software Version 16 (*p* > 0.05). Some of the measured parameters are given in Table 1.

## 3. Results

### 3.1. Radiographic Result

**Figure 2:**
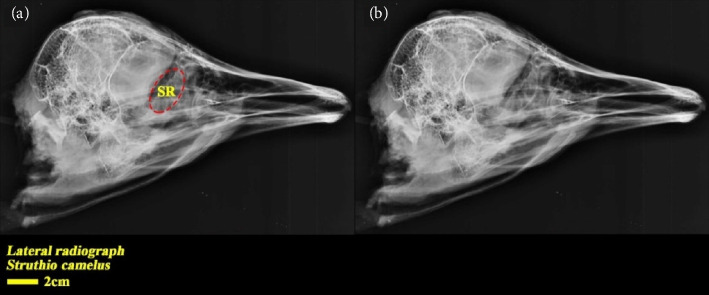
Lateral radiograph of the head of the ostrich (*Struthio camelus*). SR: scleral ring.

**Figure 3:**
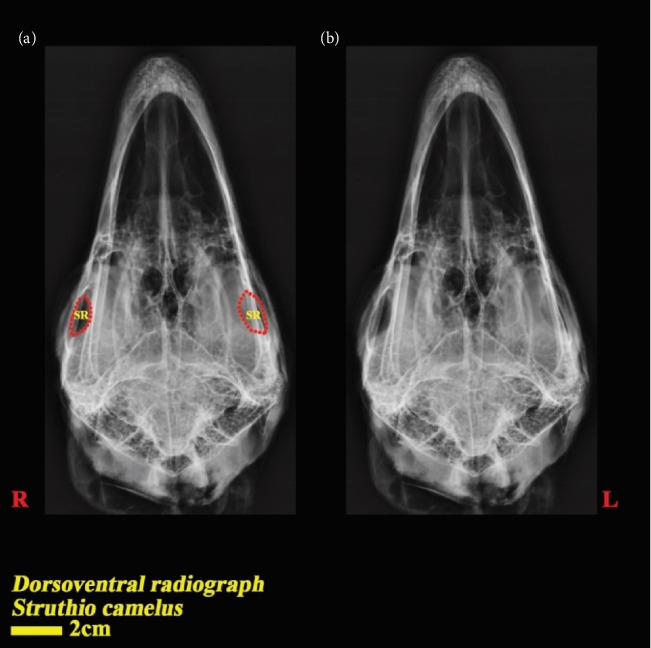
Dorsoventral radiograph of the head of the ostrich.

In the dorsoventral and lateral views, the structure of the scleral ring with bone opacity is clear and its existence can be confirmed. In the lateral view of the radiograph image, due to overlapping structures, it is difficult to examine the ring, but in the dorsoventral view, this structure is more clearly defined and distinguishable, and the exact location of this structure in the skull and the integrity of the entire structure and its opacity can be examined. In the dorsoventral view, it is clear that this structure is not connected to other parts of the skull. In radiographic images, it is not possible to separate and count the ossicles in the ring. For more detailed investigations, CT scan and micro-CT scan techniques have been used (Figures 2 and 3).

### 3.2. CT Scan Result

**Figure 4:**
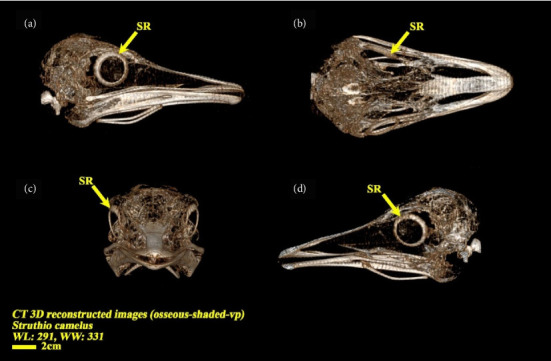
CT three-dimensional reconstructed images (osseous-shaded-vp) of the ostrich (*Struthio camelus*), showing bones of the skull, (a) lateral view, (b) dorsal view, (c) anterior view, and (d) right sagittal section view; SR, scleral ring.

**Figure 5:**
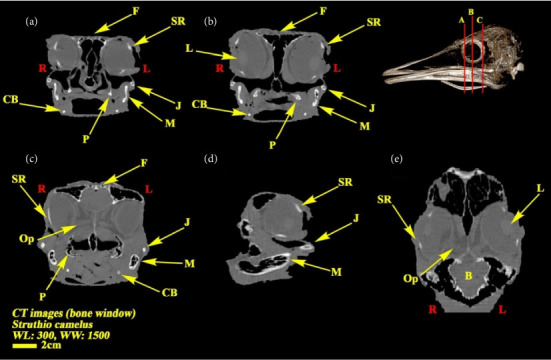
CT images of the head of the ostrich (*Struthio camelus*) ((a–c) transverse planes, (d) sagittal plane, and (e) dorsal plane). B: brain; CB: ceratobranchial bone; F: frontal bone; J: jugal bone; L: lens; M: mandible; Op: optic nerve; SR: scleral ring.

In examining the results obtained from the CT scan of the samples, the findings can be analyzed in 2D and 3D forms. In the 3D view of the skull, you can see that the scleral ring is a thin and round ring that is not connected to the skull, and it is made up of small ossicles and is located inside the eyeball, but the number of ossicles and their quality are not well known. In the 2D view, the bone structures of the skull can be clearly seen, and the scleral ring can also be seen in different views (Figures 4 and 5).

### 3.3. Micro-CT Scan Result

**Figure 6:**
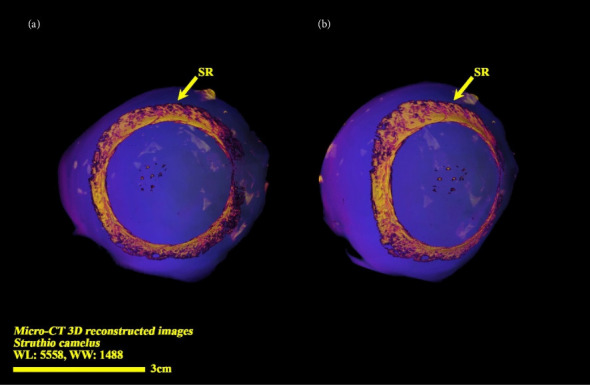
Micro-CT three-dimensional reconstructed images of the right eye ball of the ostrich (*Struthio camelus*): (a) anterior view; (b) anterolateral view; SR: scleral ring.

**Figure 7:**
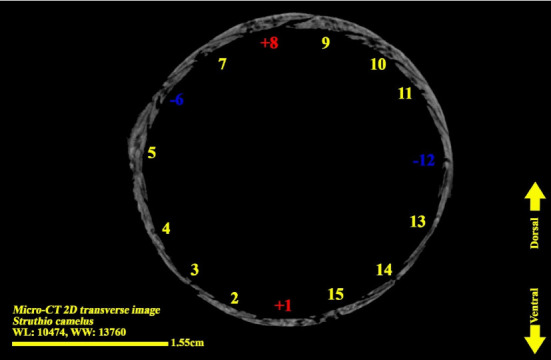
Micro-CT two-dimensional transverse image of the right eye ball of the ostrich (*Struthio camelus*) (15 ossicles); plus and minus excellent ossicles are shown.

**Figure 8:**
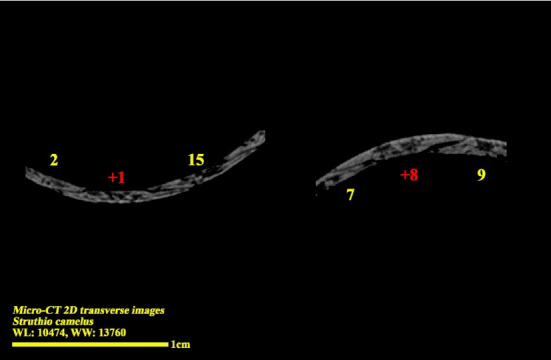
Micro-CT two-dimensional transverse images of the right eye ball of the ostrich (*Struthio camelus*) (15 ossicles); plus excellent ossicles are shown.

**Figure 9:**
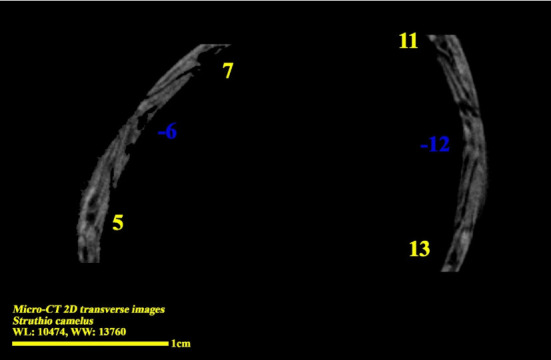
Micro-CT two-dimensional transverse images of the right eye ball of the ostrich (*Struthio camelus*) (15 ossicles); minus excellent ossicles are shown.

Micro-CT scan images are similar to CT scan, but its resolution is much higher and the structure of the ossicles inside the scleral ring can be examined more clearly. 2D and 3D images are prepared in micro-tomography. In 2D micro-CT images, the length, width, and height of each ossicle and the distance between the ossicles can be seen. The number of ossicles in right and left scleral ring of an ostrich can be different (Figures 6, 7, 8, and 9).

### 3.4. Gross Anatomical Result

**Figure 10:**
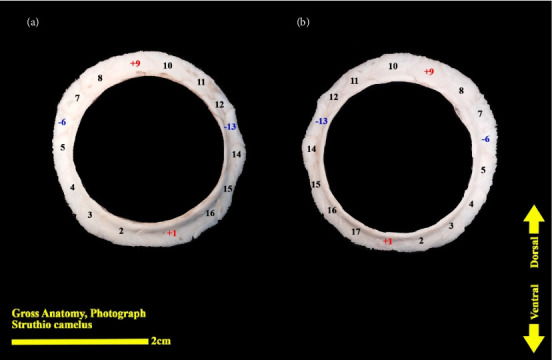
Scleral ring of the ostrich (*Struthio camelus*): (a) right scleral ring, anterior view; (b) left scleral ring, anterior view; plus and minus excellent ossicles are shown.

**Figure 11:**
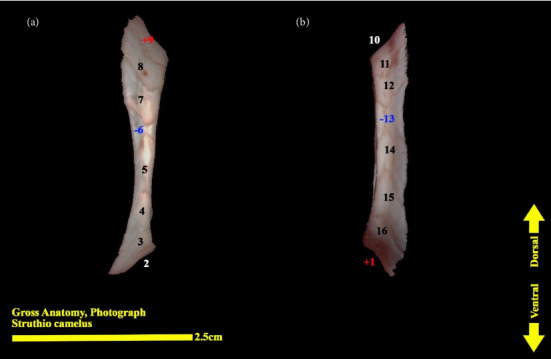
Right scleral ring of the ostrich (*Struthio camelus*): (a) lateral view; (b) medial view; plus and minus excellent ossicles are shown.

**Figure 12:**
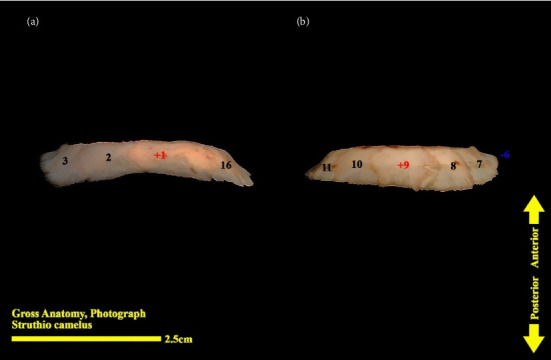
Right scleral ring of the ostrich (*Struthio camelus*): (a) ventral view; (b) dorsal view; plus and minus excellent ossicles are shown.

**Figure 13:**
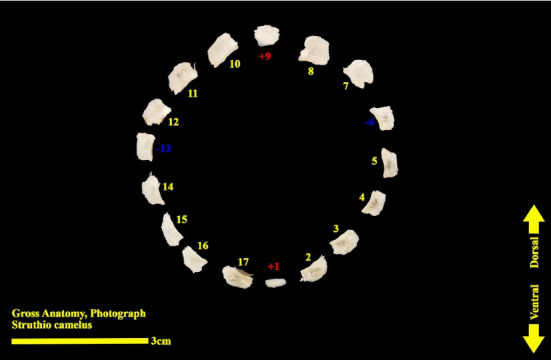
Left scleral ring of the ostrich (*Struthio camelus*); ossicles are dissected; plus and minus excellent ossicles are shown.

**Figure 14:**
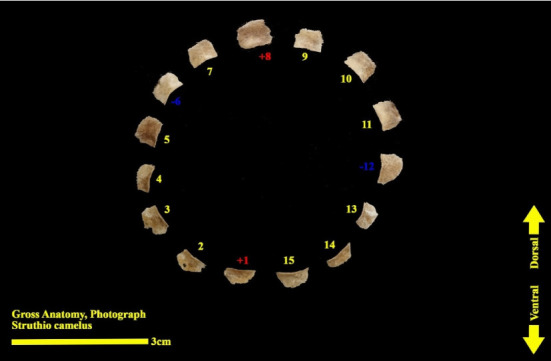
Right scleral ring of the ostrich (*Struthio camelus*) (15 ossicles); ossicles are dissected; plus and minus excellent ossicles are shown.

**Figure 15:**
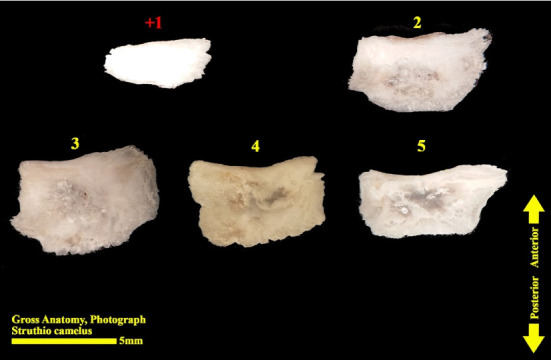
Ossicles 1–5 of the left scleral ring of the ostrich (*Struthio camelus*), external surface.

**Figure 16:**
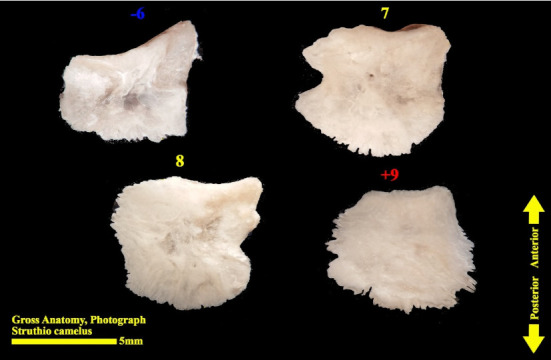
Ossicles 6–9 of the left scleral ring of the ostrich (*Struthio camelus*), external surface.

**Figure 17:**
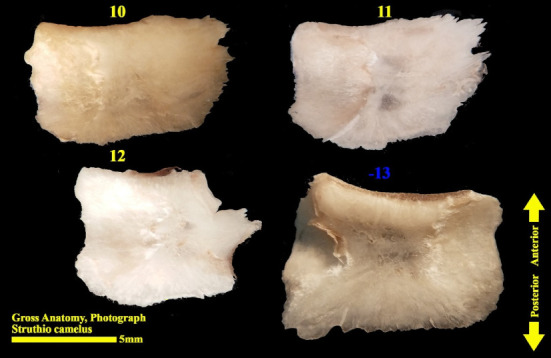
Ossicles 10–13 of the left scleral ring of the ostrich (*Struthio camelus*), external surface.

**Figure 18:**
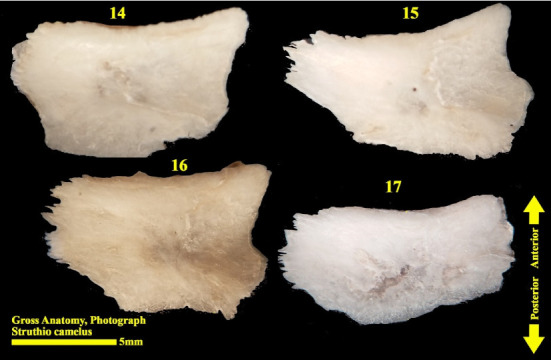
Ossicles 14–17 of the left scleral ring of the ostrich (*Struthio camelus*), external surface.

The scleral ring is seen with a semi-hyperbolic structure, the diameter of the anterior opening is greater than the posterior part, and the structure of the ring is slightly concave and depressed on the outer side and slightly convex on the inner side. Cross sections of the ring are relatively round, and its vertical and horizontal diameters are almost equal. The anterior edge has a smooth structure, but the posterior edge is quite dentate and the scleral ring does not have any joint or connection with other bony parts of the skull. The dorsolateral part of the rings is less thick, but in contrast, the ventromedial part is thicker and has more thickness. After observing the ring under the loupe, it was observed that the scleral ring in the ostrich consists of 15–17 ossicles, which have a rectangle appearance. Ossicle number one is located in the ventral part of the scleral ring, and both of its articular surfaces are placed above the two ossicles adjacent to it, so it is a plus excellent ossicle. To count the ossicles belonging to the right eye scleral ring, we move clockwise (to the lateral side) and in the rings belonging to the left eye, we move counterclockwise (to the lateral side) and toward the temporal bone. It can be seen that in the lateral part of all the scleral rings, ossicle number 6 is located, which is minus excellent ossicle, and in the dorsal area, another positive ossicle is seen, which is ossicle number 9, and in some others, number 8. We have another minus excellent ossicle which is ossicle number 12 or 13. By observing that the scleral ring has 4 excellent ossicles in all samples, we come to the conclusion that according to the existing classifications, the scleral ring in ostriches is Type A, unlike owls that have only two excellent ossicles (Figures 10, 11, 12, 13, 14, 15, 16, 17, and 18).

A 360-degree view of the 3D reconstruction of the scleral ring of the right eye of an ostrich can be seen in the short clip provided as supporting information alongside this article (Clip 1).

### 3.5. Morphometric Result

The following tables show the results of morphometric studies, and *p* < 0.05 indicates a significant difference between two parameters. The same alphabet in front of the parameters means that there is no significant difference between those parameters (Tables 2, 3, 4, and 5).

There was no significant difference between the volume of the eyeball, lens, anterior and posterior chamber, and vitreous chamber in right and left eyes (*p* > 0.05). There was no significant difference between the parameters such as anterior diameter, posterior diameter, length of scleral ring, eye ball diameter, and length and diameter of optic nerve in left and right eyes (*p* > 0.05). By measuring the thickness, length, and width of the ossicles of the scleral ring, it was determined that the excellent plus, excellent minus, and interlocking ossicles do not differ significantly in terms of size (*p* > 0.05). The ratio of eye volume to brain volume has been compared. In this study, it was observed that in ostriches, the volume of the brain is larger than one eye, and the total volume of two eyes is more than brain. Based on the results of micro-CT scan images, it was observed that 67.26% ± 6.22 of the bone tissue of each ossicle is compact bone and 32.73% ± 6.22 is cancellous (spongy) bone. To compare the thickness of the scleral ring on the medial and lateral sides of each ring, three ossicles from the medial side, ossicles No. 11, 12, and 13, with three ossicles on the lateral side, ossicles No. 5, 6, and 7, from a 15 ossicle ring in terms of thickness were compared to each other. A significant difference was observed between the measured parameters, and the thickness of the ossicles is greater on the medial side (*p* < 0.05).

## 4. Discussion

The form of the scleral ring and it ossicles can be investigated for the purpose of animal classification (especially in rare species) and the diagnosis of abnormalities. Examining the separate role of each ossicle is discussed, and no exact results have been reached yet, while the applications of the scleral ring have been well described, the most important of which is that the scleral ring protects the eye against external pressure and trauma and helps maintain the shape of the eyeball during flight. The oval eyeball of birds increases the eye's resistance to water and air pressure and protects the eyes from deformation [27].

So far, not many studies have been carried out about eye ball of ostriches and the scleral ring has not been studied in any of them. In past studies, the structure of the scleral ring in some other birds such as owls and penguins has been studied, but the scleral ossicles have not been measured in any of them. In this study, the structure of eye ball, scleral ring, and ossicles have been studied.

In the study of Monfared and Bakhtiari, which was conducted on 20 ostrich eyes, the anatomical examination of the eye components and their measurements were discussed. From the results of this study, it can be mentioned that the average vertical and horizontal length of the ostrich eye is 6.3 and 9.63 cm. The eyeball is protected by the upper, lower, and third eyelids, and the lower eyelid is wider than the upper eyelid and is mainly responsible for closing the eyes. In ostriches, like other birds, the eyelids do not have sweat or sebaceous glands, and there are small bony plates inside the eye (scleral ring) to create rigidity [35].

There are reports about fractures of the scleral ring in a variety of birds due to head trauma and its diagnosis. In 1988, Lindley et al. diagnosed a fracture in the scleral ring of the eye of *Buteo jamaicensis* using radiographic images [36]. Also, in the same year, researchers investigated the scleral bone ring in *Opisthocomus hoazin* and examined it in terms of phylogenetic connections [37].

Based on the research conducted on the phylogeny of the scleral ring in birds and fishes, they concluded that the embryonic bones in the scleral ring originated from the cartilages of the same ring with the difference that the embryonic bones in birds are dermal bones [27]. Franz Odendaal and Tamara studied the morphology of cartilaginous structures that turn into bone with the stages of chick development. It has been concluded that ossification in embryonic stages is from the inner edge of the ring to the outer edge [9].

In 2009, Lima investigated the structure of the scleral ring in some Brazilian birds, and one of the results is that in *Athene cunicularia*, the number of ossicles is 17, which is similar to some of our results in this study. In this study, Lima also examined *Rhea americana*; in this bird, the number of ossicles of the scleral ring is 15 in some samples and 16 in others, which is similar to some of our samples in this study. The number of ossicle in the right and left eye can be different in some species [27]. In the current study, it was revealed that the wall of the medial side of the ring is thicker than the lateral side, and the posterior border of the ring is sharper than the anterior border.

In studies related to 1998, it has been said that the structure of the scleral ring in some birds has a piece of bone that has never been mentioned before and the anatomical description of its structure has not been stated; this structure is called *sesamoid* bone of the sclera. In Mahecha's studies, it was mentioned that the structure of the scleral *sesamoid* bone was not seen in *Glaucidium brasilianum*, but in some birds, it has a round and small structure, in others, it is stretched, and in others, it is two-segmental [38]. In our study, no *sesamoid* bone was observed in the examinations performed on the ostrich scleral ring. The probability of the presence of *sesamoid* bone and ignoring it due to a mistake in the bone separation process is zero. If there was a structure in the size of Mahecha's report (approximately 3 mm), it would be seen in the images taken by a micro-CT scan with a 25 μm slice thickness.

According to the studies conducted by Hadden et al. in 2021 on the scleral ring of different penguin species using micro-CT scan, it has been pointed out that in *Pygoscelis papua* chicks (at the age of 10 weeks), the amount of spongy bone tissue in the ossicles is much higher than in adults. In our study, which was conducted on adult ostriches, on average, about 67% of the volume of each bone is dense bone and the remaining 33% is spongy bone [39].

In 2022, Zehtabvar et al. investigated the scleral bone ring in *Asio otus* and found the following results that the scleral bone ring in the right and left eyes is symmetrical in terms of shape, size, and number of bones forming the ring. This structure consists of 15 bones, and there was no significant difference between the parameters measured in the left and right eyes, but most of the eye parameters measured in female owls were larger than males, such as the size of the eyeball. According to the results of this study, the ratio of the volume of one eye (right eye) to the volume of the brain in the male *Asio otus* is 1.2, which is 0.81 in *Struthio camelus* [40].

As reported by Franz Odendaal et al. in 2006, the ossicles are predominantly quadrangular in shape (without uniformity). In Falconiformes, they are square or rectangular, in Psittaciformes, Columbiformes, and Gruiformes, they are trapezoidal, and in Piciformes, they are irregular and have sinuous borders [23]. In our study on ostrich, the ossicles have a rectangular appearance and the average ratio of width to length (AP) of these ossicles is 1.44, but in owls, the length of the ossicles can be twice the width.

The scleral ring is tubular in birds of prey and owls, but it is nontubular in *Gallus gallus* and penguins like ostriches [41]. In *Gallus gallus*, the number of ossicles in the left and right eyes is equal, and in all samples, there are 14, ossicles number one and nine are plus and ossicles number six and ten are minus, it means the scleral ring in *Gallus gallus* is Type A [37].

Another bird from the Galliformes order studied by Queiroz and Good was *Guttera plumifera*. In four samples of the scleral ring that was examined from this bird, it was observed that all the samples have 14 ossicles. In three samples, the scleral ring was Type A and had four excellent ossicles, but in one ring, only two excellent ossicles were seen and it was Type B. This study showed that the type of scleral ring can be different in the same species, but in our study, all samples were similar in terms of the number of excellent ossicles [37].

By comparing the results of necropsy and the results of diagnostic imaging in this study, we come to the conclusion that in living animals, without the need for euthanization and necropsy, the structure of this ring can be investigated for the purpose of animal classification (especially in rare species) and the diagnosis of abnormalities.

## 5. Conclusion

This study reveals that the structure of scleral ring in ostriches consists of 15–17 ossicles. The number of ossicles can be different in left and right eyes but the shape and size of them are same. These ossicles are almost rectangular and do not have significant difference in size. The scleral ring in this bird is not tubular.

## Figures and Tables

**Table 1:** Morphometric parameters of this study.

Parameters	Visual description
Anterior diameter (scleral ring): It was taken from 3D CT scan images and 3D micro-CT scan images. This parameter denotes the measurement of the transverse diameter of the anterior portion of the scleral ring, whereby the maximum distance is determined.	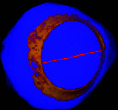

Posterior diameter (scleral ring): It was taken from 3D CT scan images and 3D micro-CT scan images. The parameter denotes the transverse diameter of the posterior segment of the scleral ring, whereby the maximum distance was recorded.	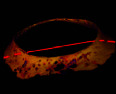

Length (scleral ring): This parameter was taken from 3D CT scan images and 3D micro-CT scan images. In this parameter, the measurement was conducted to determine the horizontal distance between the anterior and posterior borders of the scleral ring. The maximum distance was subsequently recorded.	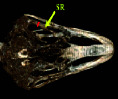

Anterior posterior diameter (eyeball): It was taken from 2D coronal CT scan and micro-CT scan images. The parameter referred to as the horizontal distance between the anterior and posterior borders of the eyeball was quantified, and the maximum distance was determined.	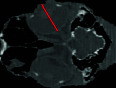

Optic nerve length: This parameter was taken from 2D coronal micro-CT scan images and 2D coronal CT scan images. The placement of the optic nerve within the eyeball was determined and its length was quantified.	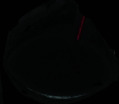

Optic nerve sheath diameter (ONSD): It was taken from 2D coronal micro-CT scan images and 2D coronal CT scan images. The caudomedial position of the eye ball was where the optic nerve was observed, and the measurement of its sheath diameter was conducted.	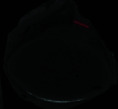

Volume of the eyeball/lens/brain: It was taken from 2D coronal, sagittal, and transverse CT scan images. In the designated segment pertaining to the quantification of volume within the Syngo MMWPVE40A software, the transverse, coronal, and sagittal views have ascertained the structural range in sections where the eyeball, lens, and brain are detected. Subsequently, the software has meticulously examined these ranges across all sections and generated a comprehensive report on the volume.	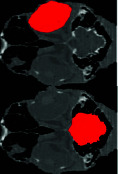

Volume of the vitreous chamber or anterior and posterior chamber: These parameters were taken by 2D coronal, sagittal, and transverse CT scan images. In the specific domain of quantifying the extent of Syngo MMWPVE40A software, in every instance where the vitreous or anterior and posterior chamber was discerned, the configuration scale was explicitly defined in the transverse, coronal, and sagittal views. Subsequently, the software processed these dimensions across all instances and the magnitude was documented.	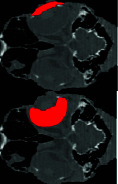

Skull length: This parameter was taken from 3D CT scan images. In this particular parameter, the measurement was taken of the horizontal distance between the anterior and posterior borders of the skull and the measurement recorded was of the greatest distance observed.	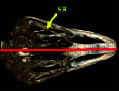

Skull height: This parameter was taken from 3D CT scan images. In this particular parameter, the measurement was taken of the horizontal distance between the dorsal and ventral borders of the skull and the measurement recorded was of the greatest distance observed.	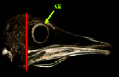

Skull width: This parameter was taken from 3D CT scan images. In this particular parameter, the measurement was taken of the horizontal distance between the right and left borders of the skull and the measurement recorded was of the greatest distance observed.	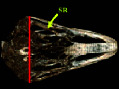

**Table 2:** Measured parameters of the scleral ring and eyeballs of the ostrich (cm).

Parameter	Right eye	Left eye
	Mean ± SD	Mean ± SD
Anterior diameter	2.49 ± 0.07a	2.53 ± 0.05a
Posterior diameter	3.42 ± 0.17b	3.34 ± 0.14b
Length of scleral ring	0.4 ± 0.07c	0.42 ± 0.04c
Eye ball diameter	3.37 ± 0.07d	3.39 ± 0.15d
Optic nerve length	0.73 ± 0.01e	0.73 ± 0.01e
Optic nerve sheath diameter	0.56 ± 0.01f	0.56 ± 0.01f

**Table 3:** Measured volumes of the eyeballs and brain of the ostrich (cm^3^).

Parameter	Right eye	Left eye
	Mean ± SD	Mean ± SD
Volume of the eye ball	29.6 ± 1.6a	31.8 ± 0.98a
Volume of the anterior and posterior chambers	5.9 ± 0.15b	6 ± 0.13b
Volume of the lens	1.07 ± 0.22c	1.09 ± 0.12c
Volume of the vitreous chamber	24.5 ± 0.98d	24.18 ± 0.98d
Volume of the brain	36.58 ± 1.45	

**Table 4:** Measured parameters of the skull of the ostrich (cm).

Parameter	Mean ± SD
Skull length	16.86 ± 1.51
Skull height	8.1 ± 0.08
Skull width	9.01 ± 0.22

**Table 5:** Measured parameters of the ossicles of the ostrich (mm).

Parameters	Width	Length (AP)	Thickness
Mean ± SD	9.64 ± 0.26a	6.11 ± 0.007b	1.17 ± 0.007c
Plus ossicles			

Mean ± SD	7.92 ± 0.16a	6.15 ± 0.26b	1.26 ± 0.31c
Minus ossicles			

Mean ± SD	8.85 ± 0.68a	6.09 ± 0.13b	1.17 ± 0.16c
Interlocking ossicles			

Mean ± SD	8.83 ± 0.74a	6.1 ± 0.13b	1.18 ± 0.16c
All ossicles			

## Data Availability

The data that support the findings of this study are available from the corresponding author upon reasonable request.

## Supporting Information

Clip 1: 360-degree view of a 3D reconstruction of the scleral ring of the right eye of an ostrich.
